# A Complex-Valued Firing-Rate Model That Approximates the Dynamics of Spiking Networks

**DOI:** 10.1371/journal.pcbi.1003301

**Published:** 2013-10-31

**Authors:** Evan S. Schaffer, Srdjan Ostojic, L. F. Abbott

**Affiliations:** 1 Department of Neuroscience, Department of Physiology and Cellular Biophysics, Columbia University College of Physicians and Surgeons, New York, New York, United States of America; 2 Group for Neural Theory, Laboratoire de Neurosciences Cognitives, INSERM U960, Ecole Normale Superieure, Paris, France; University of Pittsburgh, United States of America

## Abstract

Firing-rate models provide an attractive approach for studying large neural networks because they can be simulated rapidly and are amenable to mathematical analysis. Traditional firing-rate models assume a simple form in which the dynamics are governed by a single time constant. These models fail to replicate certain dynamic features of populations of spiking neurons, especially those involving synchronization. We present a complex-valued firing-rate model derived from an eigenfunction expansion of the Fokker-Planck equation and apply it to the linear, quadratic and exponential integrate-and-fire models. Despite being almost as simple as a traditional firing-rate description, this model can reproduce firing-rate dynamics due to partial synchronization of the action potentials in a spiking model, and it successfully predicts the transition to spike synchronization in networks of coupled excitatory and inhibitory neurons.

## Author Summary

Neuronal responses are often characterized by the rate at which action potentials are generated rather than by the timing of individual spikes. Firing-rate descriptions of neural activity are appealing because of their comparative simplicity, but it is important to develop models that faithfully approximate dynamic features arising from spiking. In particular, synchronization or partial synchronization of spikes is an important feature that cannot be described by typical firing-rate models. Here we develop a model that is nearly as simple as the simplest firing-rate models and yet can account for a number of aspects of spiking dynamics, including partial synchrony. The model matches the dynamic activity of networks of spiking neurons with surprising accuracy. By expanding the range of dynamic phenomena that can be described by simple firing-rate equations, this model should be useful in guiding intuition about and understanding of neural circuit function.

## Introduction

Descriptions of neuronal spiking in terms of firing rates are widely used for both data analysis and modeling. A firing-rate description of neural data is appealing because it is much simpler than the full raster of spikes from which it is derived. In much the same spirit, firing-rate models are useful because they provide a simpler description of neural dynamics than a large network of spiking model neurons. Although firing rates are, at best, an approximation of spiking activity, they are often a sufficient description to gain insight into how neural circuits operate. Toward these approaches, it is important to develop firing-rate models that capture as much of the dynamics of spiking networks as possible.

A number of attempts have been made to derive firing-rate models as approximations to the dynamics of a population of spiking neurons [1]–[5]. Inevitably, the resulting models involve a compromise between accuracy and simplicity. Typically, such models describe firing-rate dynamics as fluctuations around a steady-state firing rate 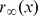. Given a constant input 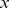, after a sufficiently long time, the firing rate will be given by 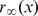.

The subtlety in constructing a firing-rate model arises in trying to describe dynamics; attempting to do so leads to two questions. First, what are the dynamics of 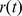 as it approaches its steady-state value 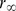
? Second, what are the dynamics in response to time-dependent input, 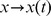
? To address the first question, it is generally assumed that the approach to the steady-state is exponential with a temporal rate-constant 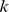 (or time constant 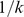), so that the time-dependent firing-rate is described by
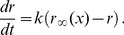
(1)We will refer to equation 1 as the classic rate model. The most straightforward approach to answering the second question, what happens when the input is time-dependent, is simply to use equation 1 with a time-dependent asymptotic rate 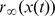, even though it was derived with a static input in mind.

**Figure 1:**
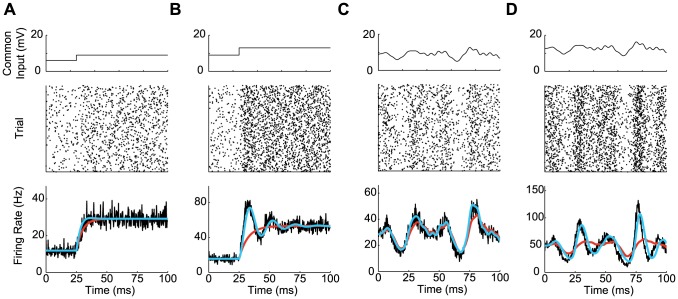
Firing-rate response of an uncoupled spiking population. **A.** Response to step in the common input current in the noise-dominated regime. **B.** Response to step in the common input in the mean-dominated regime. **C.** Response to randomly fluctuating common input in the mean-dominated regime. **D.** Response to randomly fluctuating common input in the mean-dominated regime. In A–D, top panel shows common injected current; middle panel shows spike raster for 500 trials with an EIF neuron; bottom panel shows firing rate response of 10,000 EIF neurons (black), the classic rate model (red), and the complex-valued rate model (blue). Background noise is constant, with A & C 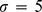
mV, B & D 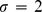
mV.

To evaluate the validity of such a rate model, an appropriate basis of comparison is the firing rate of a population of identical spiking neurons, all receiving the same common input 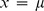 and each receiving independent ‘noise’ fluctuations with the same variance 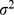. Under appropriate conditions, the classic rate model of equation 1 can provide a reasonable approximation. For example, the change in the firing rate of a population of uncoupled integrate-and-fire model neurons responding to a step change in their common input matches the results of equation 1 quite well when the independent noise dominates their dynamics (Figure 1A, red trace). However, when the dynamics of the same population of integrate-and-fire neurons is dominated by the mean of the input rather than the noise, the model neurons tend to transiently synchronize their firing in response to a step change in 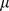, resulting in an oscillating firing rate that is not well described by equation 1 (Figure 1B, red trace). Similar results are obtained in response to more general time-dependent common inputs (Figures 1C and D). In the noise-dominated regime, these dynamics can be matched by equation 1 (Figure 1C, red trace), but when the common input dominates the noise, equation 1 fails to capture the large firing-rate fluctuations (Figure 1D, red trace), even when 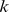 is chosen optimally (Methods). In this paper, we introduce a firing-rate model based on a generalization of equation 1 to complex numbers that can describe the firing rate in all of these cases (Figure 1, blue traces). Our focus is on describing, within a rate formulation, effects caused by partial spike synchronization that cannot be considered in conventional rate models, rather than on describing phenomena such as excitatory-inhibitory oscillations that can and have been analyzed using conventional models.

A powerful method for analyzing spiking dynamics is to use the Fokker-Planck equation to compute the probability density of membrane potential values for a population of model neurons. This approach has been used to analyze the synchrony effects we consider [6]–[8], to study the impact of synaptic dynamics [8], [9], to compute linear responses [10], [11], and to explore models of zsensory processing [7]. Simulation of a network using the Fokker-Planck approach requires integration of a partial differential equation. This can be done with reasonable computer power, and previous work has suggested ways of simplifying the Fokker-Planck analysis by approximating the results with a finite number of modes [6], [8], [12]. Our work makes use of an extreme limit of this approach and results in a model that directly describes firing rates in terms of an ordinary differential equation. A firing-rate description offers some computational advantages over the full Fokker-Planck description but, more importantly, it opens up possibilities for analytic studies based on stability analysis and mean-field approximations [13].

Classic rate models fail to describe neuronal firing when noise is insufficient to eliminate spike synchronization. The basic problem is that firing-rate dynamics are not purely exponential with a constant decay rate. The decay rate 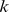 in equation 1 can depend on 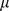 and 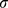. In addition, multiple exponentials and, as seen in Figure 1B, oscillatory dynamics may be required. Two previous studies of firing-rate models have addressed different aspect of this problem by retaining equation 1 but replacing the simple equality 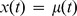 with a differential equation that relates 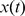 to 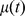 [4], [5]. Shriki et al. [4] introduced a differential equation that describes damped firing-rate oscillations such as those seen in Figure 1B, but the decay rate of these oscillations is a constant rather than changing as a function of the firing rate and spiking variability as it does in spiking models. This analysis was based on a comparison of their rate model with a conductance-based neuronal model. Ostojic & Brunel [5] computed how the relaxation time constant depends on 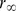 by approximating the linear response computed from the Fokker-Planck equation. This analysis did not consider an oscillatory component in the approach to the steady-state. Here, we aim to describe both the variability in the rate of decay of the firing rate to its steady-state value and the oscillations that may occur during this transition. Whereas firing-rate models are typically thought to be inappropriate outside the noise-dominated regime, we show that the resulting ‘complex-valued’ firing rate model can still describe neural dynamics well into the mean-dominated regime. Furthermore, we find that the firing rates of the different spiking models we consider – the linear (also called “leaky”), quadratic and exponential integrate-and-fire models (LIF, QIF and EIF, respectively) – can all be described using a single, and therefore general, complex-valued firing-rate model.

## Results

As outlined in the Introduction, the classic rate model is limited because it attempts to describe firing-rate dynamics using a single exponential with a fixed decay rate. The previous extensions of the classic model described in the Introduction focused on the relationship between 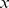 in equation 1 and 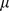, the common input to the spiking neurons being modeled. We leave 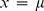, even in the time-dependent case, and focus, instead, on modifying equation 1. In general, considering dynamics composed of multiple rather than a single exponential would result in a more complicated model, but moving from a fixed to a varying decay rate is simple –- we just allow 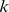 in equation 1 to be a function of 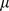 and 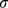. Extending the dynamics from exponential to oscillatory can also be done easily by replacing 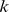 and 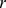 in equation 1 with complex variables that we denote as 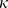 and 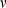, respectively. The actual firing rate is given by the real part of the complex firing rate, and thus the complex-valued rate model is defined by
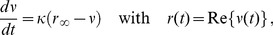
(2)where 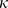 is complex-valued, and both 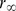 and 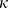 depend on 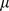 and 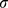 (we omit these dependencies for notational simplicity). To complete the definition of the model, we must specify the dependence of 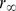 and 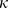 on the input parameters. The first of these, 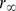, can be obtained either through first-passage time or other analytic calculations or by fitting numerical results. Here we focus on determining 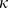 using a Fokker-Planck approach.

The spiking neuron models we study are all based on the equation
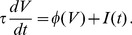
(3)We consider three types of integrate-and-fire models, determined by the form of 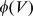. For the LIF model, 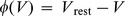; for the QIF model, 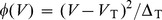; and for the EIF model, 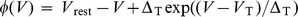. Here, 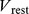, 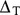 and 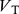 are fixed parameters. All three models generate action potentials when the membrane potential reaches a threshold value 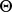 and are then reset to a potential 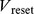. The chosen parameter values for all models are listed in the Methods. The input current we consider is of the form
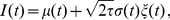
(4)where 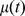 gives the mean current at time 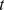, 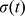 determines the trial-to-trial or neuron-to-neuron variability of the current, 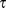 is the membrane time constant appearing in equation 3, and 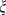 represents random white noise with first- and second-moment averages 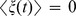 and 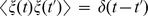. The firing rate we model is the spiking rate of this model neuron averaged over many trials with independent draws of the white noise or, equivalently, the average firing rate of a population of uncoupled neurons described by equations 3 and 4 with the noise drawn independently for each neuron. Later we consider coupled networks.

### The Fokker-Planck Approach

Firing-rate models attempt to characterize the action potentials generated by a population of spiking neurons without accounting in any way for further biophysical quantities such as the membrane potentials of the neurons. An alternative approach is to use the Fokker-Planck equation to compute the distribution of membrane potential values across the population as a function of time, and then to derive the firing rate from this distribution. This can be done by expanding the distribution in a series of modes that are eigenfunctions of the Fokker-Planck operator. In the Methods, we show that equation 2 can be derived as a two-mode approximation of the firing rate that arises from this eigenfunction expansion and that, as a result, 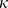 in equation 2 is the negative of the dominant nonzero eigenvalue of the Fokker-Planck operator. This provides a way to compute 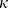 as a function of the common input and input variance, 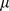 and 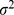. In using an input current (equation 4), which is an approximation of the Poisson input that a neuron would receive in a network in a form suitable for Fokker-Planck analysis [9], we ignore both the conductance and temporal filtering effects of synapses. The latter simplifies the Fokker-Planck analysis by avoiding dynamic variables related to synaptic transmission [8], [9].

### Determination of 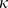


**Figure 2:**
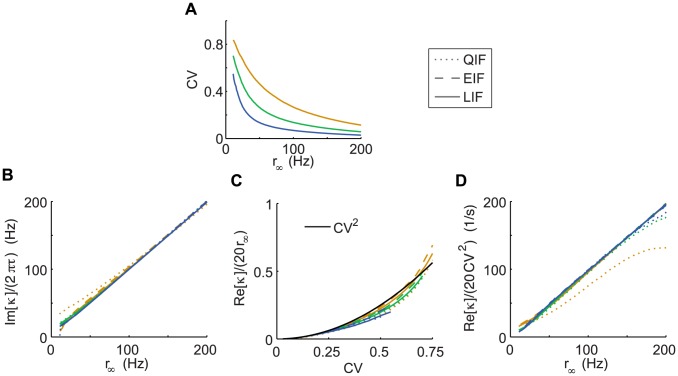
The parameter 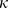 as a function of firing rate and CV. **A.** Curves through 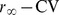 space along which values in B–D are evaluated. **B.** Imaginary part of 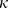 divided by 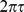. **C.** Real part of 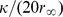 versus CV. Black line corresponds to 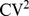. **D.** Real part of 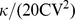 versus 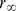. B–D show 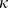 for the QIF (dotted lines), EIF (dashed lines), and LIF (solid lines), with the color indicating the corresponding line in 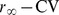 space shown in A.

The computations of the dominant nonzero eigenvalue of the Fokker-Planck operator for these models are described in the Methods, and the results are shown in Figure 2. Rather than expressing 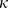 as a function of the input parameters 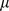 and 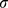, we use an equivalent parameterization in terms of the output, expressing 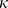 as a function of 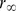 and the coefficient of variation (CV) of the spiking models. The 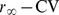 space has a one-to-one mapping with the 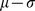 space [14], and working in this space allows us to plot results for all three neuron models on comparable axes. In Figure 2B–D, we show the imaginary and real parts of 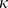 along the curves in the space of 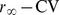 values depicted by the different colored traces in Figure 2A (these are curves of fixed 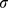 for the exponential integrate-and-fire model, in particular 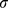 = 1, 2 and 4 mV).

The first thing apparent in Figure 2 is that the 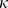 values for the models do not differ from each other very much over the range shown, although the imaginary parts separate somewhat at low 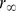 values and the real parts deviate from each other at high CV values. As shown in Figure 2B, the imaginary part of 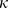 is approximately a linear function of 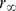 for all three integrate-and-fire models, with a slope of 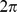. This dependence is not unexpected. Going back to Figure 1B, we note that the oscillations following the step increase in the common input are due to partial synchronization of the spike times across trials. As a result, the mean spacing between these peaks is equal to the interspike interval of the spiking model neuron, so the frequency of these oscillations is the steady-state firing rate 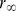.

The dependence of the real part of 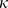 on the 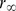 and the CV value is not as simple as that for the imaginary part but, as shown in Figure 2C, the quantity 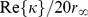 depends on the coefficient of variation in an approximately model-independent manner. For CV values less than 0.75, 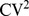 provides a good fit to the data (Figure 2C, black). Equivalently, the quantity 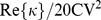 is approximately equal to 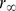 unless both 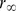 and CV are too large (Figure 2D). Putting these two pieces together and noting that 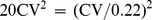, the complete complex-valued firing rate model consists of equation 2 with
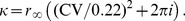
(5)For CV values near 1 and above, 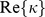 is better fit by a power-law dependence with a power greater than 2. However, in the following, we focus on the parameter range where the effects of spiking synchrony and the differences between the complex-valued and classic rate models are largest, which is the region of smaller CV values.

In evaluating differences between the integrate-and-fire models and the accuracy of our fits (Figure 2), it is important to note that the significance of such difference varies as a function of 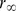 and CV. In particular, for large values of either of these parameters, the dynamics become fast, that is, 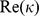 becomes large. We are primarily interested in matching the dynamics of the complex-valued rate model to the spiking models over a fairly low frequency range. For this purpose, it makes little difference whether the decay rate of the transients matches exactly, as long as it is fast. For this reason, discrepancies are less concerning when 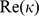 is large.

The results summarized in equation 5 come from a Fokker-Planck analysis assuming time-independent input variables 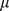 and 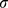 (Methods). If we redo the analysis allowing for time-dependence in these variables, additional terms spoil the correspondence between the truncated Fokker-Planck and rate-model approaches [12]. However, comparison with populations of integrate-and-fire model neurons convinced us that these terms are small unless the input parameters vary extremely rapidly over wide ranges. Thus, we follow the step often taken in deriving the classic firing-rate model and discussed in the Introduction, which is to use the model defined by equations 2 and 5, even when 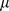 and 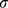 depend on time, simply by using the time-dependent values in these equations.

Classic firing-rate models are completely specified by the function 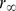 and constant 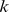. The complex-valued rate model is similarly specified by 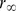 and the CV value. In the coupled networks that we consider in a following section, CV may change over time, but it can be determined easily as a function of the network activity as the network state evolves.

### The Complex-Valued Rate Model Reproduces the Firing-Rates of Spiking Neurons

We have already shown in Figure 1 that the complex-valued rate model does much better than the classic firing-rate model at describing responses in the mean-dominated regime, and it matches the performance of the classic model in noise-dominated cases. We now extend these results by studying how faithfully the complex-valued model predicts the firing rate of a neural population receiving dynamic input overlaid on different levels of background noise.

**Figure 3:**
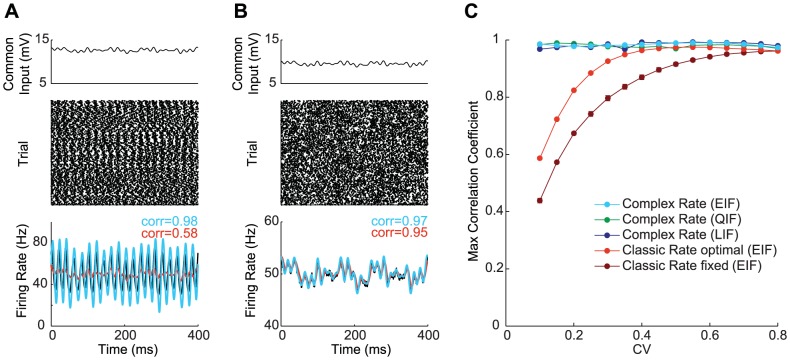
Rate model accuracy as a function of input noise. The response of each rate model is compared to a spiking population receiving an input with fluctuating common term and constant variance. The common input is composed of a baseline level and a fluctuating component composed of equal-amplitude sinusoidal oscillations with random phases and frequencies of 61, 50, 33, 13.1, and 7.9 Hz. **A–B.** Response of EIF population and both rate models to input with a CV of either 0.1 (A) or 0.8 (B). Top, middle, and bottom panels are as described in Figure 1. **C.** For each spiking model and each CV value, the maximum of the shifted correlation coefficient is computed between the trial-averaged firing rate of the spiking population and each rate model. The trial-averaged firing rate of a spiking population is computed from 300 repetitions of the same common input and different instantiations of noise. Each point in C represents the mean 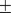 standard error of 10 different instantiations of the random phase shifts in the common input. In most cases, error bars are smaller than the marker. The maximal shifted correlation coefficient between the complex-valued rate model and the EIF, QIF, and LIF are shown in cyan, green, and blue, respectively. The same comparisons between the EIF and the classic rate model either optimized for each CV value or just to CV = 0.8 are shown in red and dark red, respectively. Classic rate model comparisons to the LIF and QIF produce similar results but are omitted for clarity.

We compare the firing rate of a population of either EIF, LIF, or QIF neurons to the classic and complex-valued rate models responding to an input of the form of equation 4, with a time-dependent common term and a range of time-independent variances. For every level of noise considered, we determine and use the optimal value of 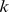 for the classic rate model (Methods), whereas we use equation 5 for the complex-valued model throughout. Figures 3A and 3B illustrate the responses at CV = 0.1 and CV = 0.8, respectively, of a population of EIF neurons, the complex-valued rate model, and an optimally fit classic rate model. When the level of noise is low, fluctuations in the common input can generate much larger fluctuations in the population firing rate than the classic model predicts, but the complex-valued model accurately reproduces the response (Figure 3A). The large firing-rate fluctuations arise in the EIF model from resonant dynamics due to partial synchronization. As we show below, the complex-valued rate model captures this resonant behavior, whereas the classic model does not. In the presence of higher noise, common input fluctuations of the same amplitude produce smaller firing-rate fluctuations and, as a result, both the complex-valued model and the optimally chosen classic rate model reproduce the response almost perfectly (Figure 3B).

We quantify the agreement between the activity of the spiking models and the complex-valued rate model using a shifted correlation coefficient. This is based on computing the cross-correlation between the firing rate of an integrate-and-fire population and that for the complex-valued rate model, but we allow for a small shift between the times at which these two rates are compared. As stated previously, we are primarily interested in matching dynamics over relatively slow timescales. Because of this, small temporal shifts are inconsequential. We therefore compute the correlation coefficient between these two rates at the shift that maximizes it. Figure 3C shows the maximal shifted correlation coefficient between each integrate-and-fire population and the complex-valued rate model. Also shown is the maximal shifted correlation coefficient between the EIF population and two different versions of the classic rate model – one in which 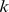 is re-optimized for each choice of CV (Figure 3C, red), and one in which 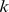 is fixed at a single value (the value optimal for CV = 0.8; Figure 3C, dark red). As illustrated in Figure 3C, performance of the classic rate model declines rapidly as the baseline CV decreases, whereas the complex-valued rate model faithfully approximates the neural population dynamics of all three neuron models across the full range of noise levels. The two models have similar accuracy for higher CV values.

### The Frequency Response of the Complex-Valued Rate Model

In the previous section, we suggested that the better performance of the complex-valued rate model compared to the classic model is due to its ability to capture resonant behavior in the underlying integrate-and-fire model dynamics. To study this further, we computed the linear response properties of the three integrate-and-fire models and compared them to the linear response of the complex-valued rate model. In particular, we considered the responses of these models to an oscillating common input 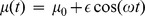 and computed them to first-order in 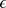. To this accuracy, the firing rate can be written as 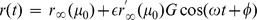, where the prime denotes a derivative. The linear response is defined by the gain 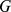 and phase 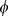, expressed as functions of the frequency 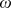. The linear response of the classic firing-rate model is just that of a low-pass filter with 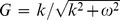 and 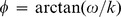, which clearly exhibits no resonant behavior.

**Figure 4:**
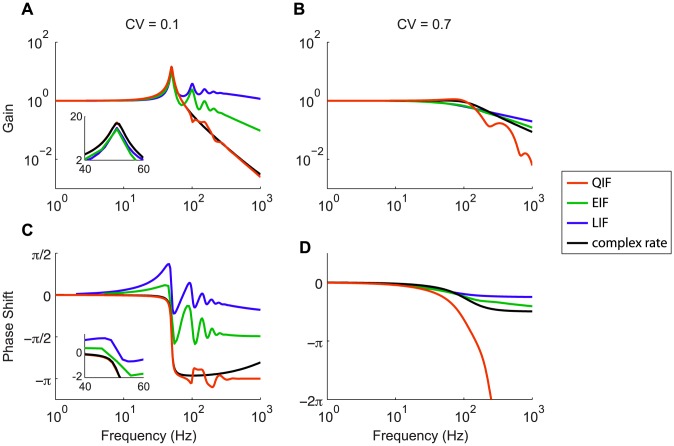
Comparison of the linear response of the complex-valued rate model and integrate-and-fire models. **A–B.** Gain and **C–D.** phase of the linear response of the QIF (red), EIF (green), LIF (blue) and complex-valued rate (black) models. **A.** and **C.** Baseline coefficient of variation of 0.1. **B.** and **D.** Baseline coefficient of variation of 0.7. Insets in A and C show gain and phase, respectively, of response near the resonant frequency of 50 Hz. In all cases, the baseline firing rate was 50 Hz.

The linear response of the complex-valued rate model is given by (Methods) 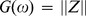 and 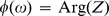, where
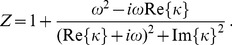
(6)The results shown in Figure 4 are based on using the steady-state rate 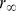 of the EIF model to compute 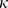, but the results are quite insensitive to which neuron model is used to define 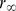.

At sufficiently low noise levels, all three spiking integrate-and-fire models have a resonance at a frequency equal to their steady-state firing rate (Figure 4A and 4C). These models exhibit similar behavior for frequencies below the first resonance peak, but their high-frequency responses differ, as has been noted previously [15]. The complex-valued rate model matches these responses fairly well below and up to the resonant frequency, and then, at higher input frequencies, matches the QIF model best over the range of frequencies shown in Figure 4. However, in the high-frequency limit, the gain of the complex-valued rate model scales as 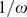, which matches the frequency response of the EIF model [15]. At high noise levels, the resonance peaks disappear, and all three spiking neuron models behave roughly as low-pass filters (Figure 4B and 4D), as do the complex-valued and classic rate models.

For the complex-valued rate model, the frequency dependence of the linear response to 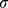 is identical to the response to 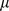 (Equation 6, but in the equation for r(t), 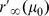 is replaced by the gain with respect to 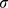). As with modulations in 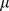, the response of the complex-valued rate model to modulations in 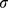 will match the response of spiking neurons at low frequencies, around the resonance, and diverge from it at high frequencies.

As shown in the insets of Figures 4A and 4C, the complex-valued rate model provides a reasonable approximation of the gain and phase of the response near the primary resonance peak for all three spiking models. As shown by Brunel & Hansel [16], network stability is fully characterized by properties of the linear response function. The similarity we see in the linear response therefore suggests that a network of complex-valued rate units should have stability properties similar to a network of integrate-and-fire neurons. We examine this in the following section.

### Excitatory-Inhibitory Networks

**Figure 5:**
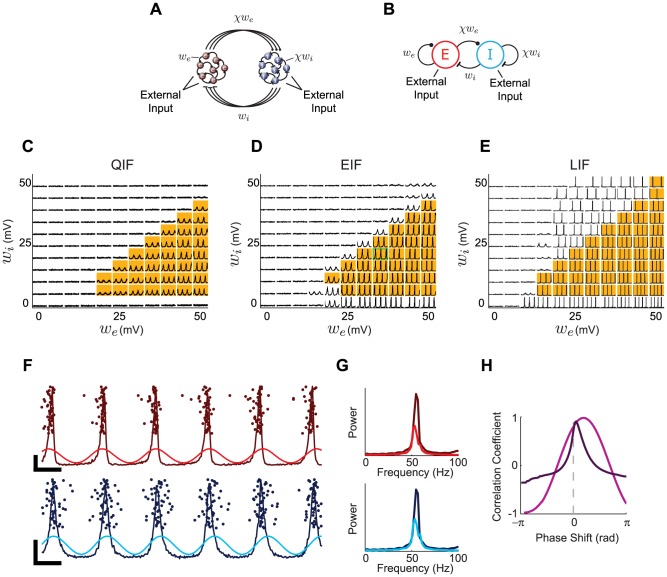
Comparison of the phase portraits of excitatory-inhibitory networks. **A.** Architecture of the large network of spiking neurons. **B.** Architecture of the network of two complex-valued firing-rate units. **C–E.** A sparse, randomly-connected network of QIF, EIF, or LIF neurons, respectively. For each connection strength, 50 ms of the firing rate of the excitatory population is shown in black. Stability diagram of the corresponding two-unit complex-valued rate-model network is superimposed on each panel, where orange indicates a stable limit-cycle, and white a stable fixed-point. A constant external input was also included with mean 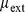 and variance 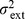 set to produce a baseline firing rate of 50 Hz and a CV of 0.1 when 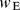 and 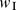 were zero. **F.** Sample excitatory (top, red) and inhibitory (bottom, blue) dynamics from both the spiking (dark) and rate (light) EIF networks with 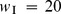 and 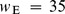 (green square in D). An exemplary spike raster of 50 neurons from each population (excitatory/inhibitory, respectively) is overlaid on the firing rate curves of both networks. Horizontal scale bar = 10 ms. Vertical scale bar = 10 Hz. **G.** Power spectra of excitatory (top) and inhibitory (bottom) units from both networks, with spiking network in darker shades and rate network in lighter shades, as in F. Both networks have a dominant frequency near 50 Hz. Curves represent mean power spectra from all parameters in D for which both networks are oscillatory (standard error comparable to line width). **H.** Cross-correlation between excitatory and inhibitory units for EIF spiking (dark purple) and rate (light purple) networks. Both networks exhibit maximal correlation at a small positive phase shift, indicating that inhibitory oscillations follow closely behind excitatory oscillations. As in G, curves represent means over all parameters producing oscillations in D, with standard error smaller than line width.

Thus far, we have shown that the complex-valued rate model can reproduce the responses of uncoupled populations of spiking neurons, but the real interest is, of course, in coupled networks. To extend our results to this case, we consider two populations of neurons, one excitatory and one inhibitory. Networks of excitatory and inhibitory neurons have been a fruitful focus of study in both rate [1], [13], [17]–[19] and spiking versions [12], [20]–[23]. In the networks we consider, the excitatory and inhibitory synaptic connections onto excitatory neurons have strengths 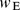 and 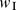, respectively. Excitatory and inhibitory connections onto inhibitory neurons have strengths 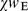 and 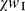, respectively (Figure 5). We keep 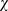 fixed at the value 0.5 and scan over different values of 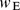 and 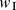. We construct both spiking networks and firing-rate networks and compare their activities.

The spiking networks we study are large, randomly-connected networks of 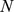 excitatory and 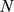 inhibitory neurons, either QIF, EIF, or LIF (Figure 5A). The connectivity is sparse, so that each excitatory (inhibitory) neuron receives 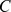 excitatory synapses of equal amplitude 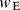 (or 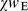 for inhibitory neurons) and 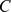 inhibitory connections of equal amplitude 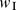 (or 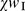 for inhibitory neurons), where 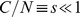. For simplicity and to match what we assumed in the Fokker-Planck analysis underlying the complex-valued rate model, we ignore the dynamics of synaptic transmission, so that at the time of a presynaptic spike the membrane potential of the postsynaptic neuron is instantaneously augmented by an amount equal to the connection strength of the synapse. Thus, 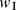 and 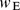 represent the integral of a synaptic current and have units of volts 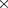 seconds. We include a white-noise external input current with mean and variance chosen so that the neurons have a baseline firing rate of 50 Hz and a CV of 0.1 in the absence of connectivity. The external noise provides additional stability to the simulations, but including it is not critical. We take 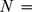
5,000 and 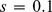.

We describe each population of the excitatory-inhibitory spiking network by one complex-valued rate model given by equations 2 and 5. The functions 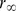 and 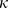 for the excitatory and inhibitory rate models are functions of the means and variances of the currents into these two types of neurons, labeled 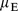, 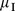, 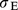 and 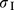. The networks are coupled through the dependence of these variables on both firing rates (Figure 5B). We also include an external source of current to each neuron with mean 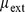 and variance 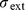. The mean and variance of the recurrent input in the spiking network can be calculated in terms of the firing rates and synaptic strengths [20], [24]. The mean inputs into the two neuron types are
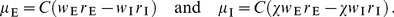
(7)The corresponding variances are
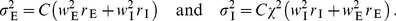
(8)From these, we compute 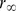 and CV by interpolating from a table of values recorded for each of the spiking models.

The firing rates of the excitatory population for each of the integrate-and-fire model types (QIF, EIF, and LIF) are shown in Figure 5C–E. Two types of behavior are evident in all three spiking neuron models. When 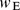 is relatively small and 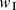 large, the neurons fire asynchronously at a constant rate. Larger values of 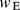 or smaller values 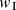 destabilize the asynchronous state causing a transition along a fairly well-defined line, to more synchronous firing with large spikes in the population firing rate. We asked whether the complex-valued rate model could predict these transitions as a function of 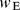 and 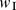.

The stability of the asynchronous state, which is the state with constant firing rates in the complex-valued model, can be computed analytically using standard procedures (Methods). The regions where the asynchronous, constant-firing-rate state is unstable are shown in orange in Figure 5C–E, overlaid on the results of the spiking network simulations. Within these regions the firing rates predicted by the complex-valued model oscillate. For the QIF, the transition boundary predicted by the rate model analytics is remarkably accurate (Figure 5C, compare orange boxes to synchronous activity). For the EIF (Figure 5D) and LIF (Figure 5E), the complex-valued model provides a fair approximation of the transitions, although with less accuracy than for the QIF. For example, the complex-valued model predicts stable asynchrony for a purely excitatory network of QIF, EIF, or LIF units (along the horizontal axes in Figure 5, but this is only true for the QIF model. Nevertheless, the transition between asynchronous and partially synchronous firing in all three spiking models can be predicted fairly well on the basis of a purely analytic calculation using the complex-valued rate model.

The oscillations seen within the orange regions in Figure 5 arise from spike synchronization; they are not the reciprocal oscillations between the firing rates of excitatory and inhibitory populations that have been analyzed in previous rate models [1], [25]. Two features of the dynamics of both the spiking and rate networks make this distinction apparent. First, the oscillation frequency in both networks is tightly tied to the baseline firing rate (Figure 5F and 5G), as it must be for oscillations due to spiking resonance; this is not a property of excitatory-inhibitory oscillations. Second, the rates of the excitatory and inhibitory units in both networks oscillate in phase (Figure 5F and 5H), rather than out of phase, as occurs in excitatory-inhibitory oscillations. Furthermore, we constructed an excitatory-inhibitory network with the architecture of Figure 5B, but built with classic firing-rate units and found that such a network is never oscillatory over the parameter range shown in Figure 5.

Given that the oscillations we report are not due to excitatory-inhibitory alternation, we might ask whether inhibition is needed at all. Indeed, the exponential and linear integrate-and-fire models can oscillate when the inhibitory weight is 0 (Figure 5D & E), although the quadratic model (Figure 5C) and the complex-valued rate model (Figure 5C–E) cannot. Using the approach of Brunel and Hansel [16], the lack of oscillations in the purely excitatory complex-valued rate model can be understood by examining the linear response in Figure 4. A self-consistent solution requires the phase shift to equal a multiple of 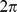 in a region where the gain is greater than 1 [16]. However, as seen in Figure 4, the phase shift of the complex-valued rate model is between 0 and 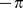, so a single complex-valued rate unit with excitatory feedback cannot generate stable oscillations. However, as seen in Figure 5, adding a small amount of inhibition lifts this restriction.

## Discussion

We have presented a simple firing-rate model that captures effects caused by synchrony in networks of spiking neurons and provides a general framework to describe neural dynamics. The model, which applies generally to the class of integrate-and-fire-type spiking models, is based on a two-mode approximation of the Fokker-Planck equation. A number of researchers [6], [8], [12] have noted that a small number of modes tend to dominate Fokker-Planck dynamics. Our approach is an extreme example of this approximation, keeping only the first non-static mode (see also Ostojic, et al. [26]). As a result, this approach should fail when other modes contribute appreciably to rate dynamics. In general, the contribution of additional modes tends to increase with the noise amplitude. The high-noise regime is also where our approximation of the dominant nonzero eigenvalue is least accurate. Nevertheless, the complex-valued rate model, like the classic model, actually performs well in this regime. This is because firing-rate dynamics are fast when the noise level is high, so although multiple modes may be involved, they are all fast. The good performance of the classic rate model at high-noise levels is similarly fortuitous. For example, the dominant nonzero eigenvalue for the QIF model is always complex, no matter how much noise is included. The classic rate model can approximate QIF rates at high noise because the resulting oscillations decay so quickly that ignoring them introduces minimal error. It should be stressed, however, that this only works if a large value of 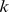 is used in the rate model when the CV gets small, and this requires a dynamically changing 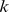 in the classic rate model.

A number of limitations of our model should be acknowledged. First, the model is only valid in the range of high input rates where the Fokker-Planck approach is applicable. Second, we have ignored synaptic dynamics, which can certainly play an important role in the dynamics of network firing rates [8], [9], [25]. Third, although the linear frequency response of the model matches that of the EIF model at very high frequencies, it matches that of the QIF model over the range relevant in most applications. This means that some high-frequency oscillations that can be achieved by networks of LIF neurons [25] may not be reproduced by our model.

The range of noise values over which our complex-valued rate model performs well depends on whether one desires quantitative or qualitative accuracy. Higher noise in the input to a spiking population tends to lead to smoother dynamics, which are more easily matched by any firing-rate model, and our model is no exception. However, as described above, smoother dynamics also tend to have non-negligible contributions from a larger number of modes, leading to a degradation in the quantitative accuracy of our model. Summarizing these constraints, the complex-valued rate model achieves quantitative accuracy describing spiking dynamics with CVs between approximately 0.1 and 0.7. For qualitative accuracy, on the other hand, the model performs well for any CV greater than 0.1.

The two key novel aspects of the complex-valued rate model are that it relates to spiking models in a general model-independent manner, and that it continues to perform well in the low-noise regime. The generality of 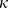 quantifies the intuition that on the timescales of interest for typical firing rate dynamics, different spiking models actually behave very similarly. Thus, when studying such dynamics, the particular choice of spiking model is inconsequential. Because the complex-valued rate model performs well not just in the high-noise regime where rate models typically operate, but also far into the low-noise regime, the model can describe oscillations generated in the underlying spiking models by partial spike synchronization. This is true whether they arise from sudden changes in the input or due to interactions with other neurons in a network. Firing-rate oscillations due to spiking synchrony have been observed in a variety of sensory systems [27]–[30]. This transient synchrony appears to be critical for the propagation of information from the thalamus to the cortex [30]. Indications of the initial synchronous burst in thalamus appear to be present in cortex as well, although at a diminished level [31]. This is presumably because noise levels in cortex tend to be high (see, for example, London, et al. [32]), but they drop significantly in the presence of sensory input [33]. Thus, accounting for transient synchrony, which the complex-valued rate model can do, is likely to be important for describing sensory responses.

## Materials and Methods

### Firing-Rate Dynamics from the Fokker-Planck Equation

The membrane potential probability density 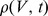 for a population of integrate-and-fire neurons described by equation 3 with the input described by equation 4 evolves in time according to the Fokker-Planck equation
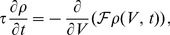
(9)with the flux operator
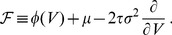
(10)


The firing rate 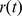 is given by the flux evaluated at the threshold 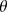,
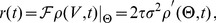
(11)with the prime denoting a voltage-derivative 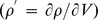. In the second equality of equation 11, we have used the fact that 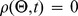. For the QIF and EIF models, the true threshold for spike generation is at infinity, so for these models the expressions in equation 11 should be evaluated in the limit 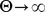.

After crossing threshold, the membrane potential is reset to 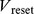, which creates a discontinuity in the flux at this point:
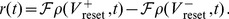
(12)


The threshold is an absorbing barrier, so
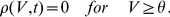
(13)Finally, because 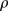 must be a continuous function of 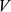,
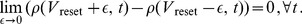
(14)


To begin, we consider the case when 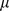 and 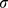 are independent of time. The membrane potential density can be expanded in a series of eigenfunctions of the Fokker-Planck operator [12],
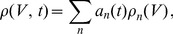
(15)where the eigenfunctions and corresponding eigenvalues are defined by
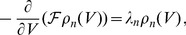
(16)and the coefficients obey
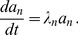
(17)The eigenvalues 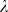 are functions of 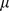 and 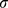. Because 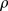 is a probability density, one of these eigenvalues, which we will label as 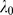, is equal to zero, and all the other eigenvalues have negative real parts. We label as 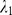 the eigenvalue with the least negative (but nonzero) real part. If 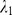 is complex, there are a pair of such eigenvalues, which are complex conjugates of each other. Either of these can be defined to be 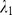, with the other 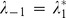.

The firing rate, expanded in terms of these eigenfunctions, is
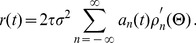
(18)Because 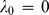, the mode with 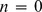 describes the steady-state properties of the neural population, and, normalizing the integral of 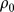 to 1 sets 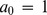, so the steady-state firing rate is given by
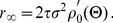
(19)The approximation that we use to derive the complex-valued rate model is to keep only this mode and the mode corresponding to 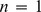, or to 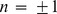 if 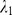 is complex. Thus, we write
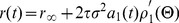
(20)if 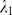 is real, or
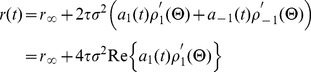
(21)if 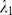 is complex. These equations can be written as
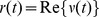
(22)if we define 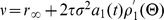 in the case of real 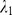, and 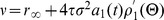 for complex 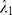. Using equation 17, it is easy to see that, in either case,
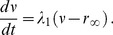
(23)This is equation 2 with, as discussed in the text, 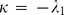.

We derived equation 23 assuming time-independent input variables 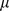 and 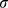. When these variables depend on time, additional terms enter into equation 17 [12] and hence into equation 23. As mentioned in the text, from various studies we concluded that these terms are typically small enough to be ignored.

### Parameters of the Integrate-and-Fire Models

The parameters used for each of the three neuron models considered are listed in Table 1.

**Table 1:** Parameters of the integrate-and-fire models.

	QIF	EIF	LIF
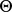 (mV)	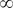	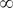	20
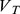 (mV)	0	10	-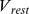 (mV)	-	0	0
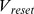 (mV)	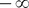	3	10
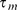 (ms)	10	10	10
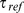 (ms)	0	2	0
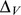 (mV)	10	1	-

### Computing the Fokker-Planck Eigenvalues for the LIF, QIF and EIF Models

For the EIF model, we compute 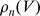 numerically by integrating the Fokker-Planck equation [11]. The eigenvalues 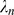 correspond to the values of 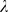 such that 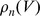 satisfies the boundary conditions equations 11–14, which determine a characteristic equation for the eigenvalues. This characteristic equation is solved numerically using a Newton-Raphson method. A similar method was followed in Ostojic [34]. For the LIF model, the functions 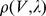 satisfying equation 16 can be computed analytically for arbitrary 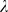 [20].

To compute the eigenvalues of the QIF, we take advantage of the fact that this model can be transformed into a phase model known as the theta model [35]. The resulting Fokker-Planck equation has periodic boundary conditions, so it can then be expanded in a Fourier series, as has been shown by Kanamaru & Aihara [36]. Keeping around 100 terms in this expansion provides an efficient way to compute the desired eigenfunctions and eigenvalues.

### Fitting 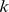 of the Classic Rate Model

We define the optimal 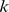 of the classic rate model for a given choice of 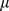 and 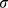 as that which best approximates the dynamics of a population of spiking neurons when 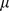 is changed from a lower value to the desired value. For a given choice of 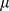 and 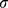, we average 100 repetitions of the simulated spiking population dynamics. For this piecewise-constant input, we can determine the response of the classic rate model analytically. Finally, we compute the optimal 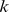 for the given parameter values by minimizing the least-squared difference between the spiking population firing rate and the computed rate-model response.

### Computing the Linear Response

For the LIF, QIF and EIF models, the linear response can be computed using the methods described in Brunel & Hakim [20] and [5], [10], [26]. For the LIF model, the linear response can be computed analytically [10], [20]. For the EIF model, the linear response is computed by integrating the Fokker-Planck equation numerically [11]. For the QIF model, the linear response can be computed by transforming to the phase representation and expanding in a Fourier series, as described above to compute the eigenvalues [36].

The linear response of the complex-valued rate model can be computed by separating the complex firing rate into its real and imaginary components, 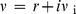, which satisfy
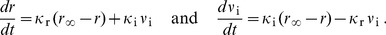
(24)We consider an input with a fixed 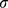 and 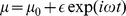 and compute all quantities to first-order in 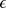. In this approximation, 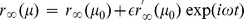, where the prime denotes a derivative. Calculating 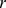 to first-order in 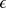 is straightforward and gives
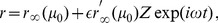
(25)with 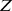 given by equation 6.

### Computing the Stable States of Excitatory-Inhibitory Networks

Stable asynchrony in the spiking network is analogous to a stable fixed point in the firing rate network and, similarly, stable synchrony in the spiking network is analogous to a stable limit cycle in the firing rate network. We calculated the stability of the fixed point in the firing-rate network. Instability of the fixed point results in the system finding a limit cycle.

Stability of a fixed point in the rate model is assessed by linearizing the dynamics around this fixed point. This involves taking derivatives of the right side of equation 2 with respect to the real and imaginary parts of the complex rate, 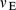 and 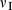. The resulting Jacobian matrix is
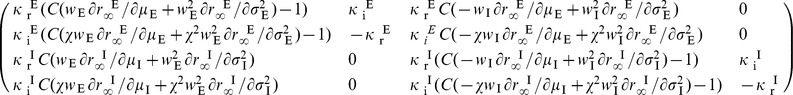



The requirement for stability is that the real part of the eigenvalues of the Jacobian matrix are negative. These eigenvalues can be computed easily for a given choice of parameters, yielding a stability diagram of stable and unstable parameter regimes, separated by a bifurcation line where the real part of either eigenvalue becomes positive.
